# Odontogenic, atypical skull-base osteomyelitis: diagnostic pitfalls and therapeutic insights—a case report and mini-review

**DOI:** 10.3389/froh.2026.1789196

**Published:** 2026-04-22

**Authors:** Parekejiang Pataer, Chen-xi Li, Yan Chen, Ya-qi Niu, Lei Yan, Zhong-cheng Gong

**Affiliations:** 1 Department of Oral and Maxillofacial Oncology & Surgery, School/Hospital of Stomatology, The First Affiliated Hospital of Xinjiang Medical University, National Clinical Medical Research Institute, Stomatological Research Institute of Xinjiang Uygur Autonomous Region, Urumqi, China; 2 Guangdong Provincial Key Laboratory of Stomatology, Guanghua School of Stomatology, Sun Yat-sen University, Hospital of Stomatology, Guangzhou, China; 3 Jiangsu Province Engineering Research Center of Stomatological Translational Medicine, Affiliated Stomatological Hospital of Nanjing Medical University, Nanjing, China; 4 Division of Pediatric Dentistry and Preventive Dentistry, School/Hospital of Stomatology, The First Affiliated Hospital of Xinjiang Medical University, Dental Medicine Institute of Xinjiang Uygur Autonomous Region, Urumqi, China; 5 Department of Dental Emergency, School/Hospital of Stomatology, The First Affiliated Hospital of Xinjiang Medical University, National Clinical Medical Research Institute, Urumqi, China; 6 Department of Anesthesiology, People’s Hospital of Xinjiang Uygur Autonomous Region, Urumqi, China; 7 College of General Practice, Xinjiang Medical University, Urumqi, China

**Keywords:** atypical, clinical management, magnetic resonance imaging, misdiagnosis, skull-base osteomyelitis

## Abstract

Skull-base osteomyelitis (SBO) represents a potentially fatal infectious condition that poses significant diagnostic and therapeutic challenges. With demographic shifts toward an older population and rising prevalence of comorbidities, physicians are encountering this complex disease with greater frequency. Current clinical practice lacks standardized protocols for both initial diagnosis and subsequent monitoring of disease progression. The present case analysis aims to provide valuable insights to guide medical practitioners in developing individualized treatment strategies for affected patients. Magnetic resonance imaging techniques demonstrate superior sensitivity in identifying early-stage bone marrow edema and soft tissue diffusion abnormalities. Current clinical evidence indicates that comprehensive antibiotic therapy with adequate dosing, when combined with prompt surgical intervention (including drainage or debridement procedures) and adjunctive hyperbaric oxygen treatment, leads to substantially improved clinical outcomes. The implementation of evidence-based early diagnostic approaches, facilitated by multidisciplinary team collaboration, has been shown to minimize diagnostic errors and decrease the likelihood of disease recurrence. In cases involving patients presenting with persistent craniofacial pain or neurological deficits, clinicians should maintain a high vigilance regarding the possibility for this condition. The implementation of a comprehensive diagnostic and therapeutic protocol incorporating advanced imaging assessment, microbial identification, and multidisciplinary case review, along with structured long-term monitoring, has been recommended to optimize patient management and improve clinical treatment outcomes.

## 1 Introduction

Skull-base osteomyelitis (SBO) typically develops as a secondary infection following otologic conditions, particularly in immunocompromised patients with underlying comorbidities such as diabetes mellitus, with elderly populations being particularly vulnerable to this severe complication (1). Atypical osteomyelitis of skull base, also referred to as central SBO, represents an emerging condition which is a clinically significant yet uncommon infectious illness with potentially severe consequences (2). This distinct pathological entity has gained increasing recognition in recent medical literature due to its diagnostic challenges and substantial morbidity. Central or atypical SBO primarily affects the regions including sphenoid, occipital bone, and clivus (2, 3). It lacks overt signs of external auditory canal involvement. This condition typically originates from occult infections in the deep facial compartments, including the masticator space, sinonasal cavities, oropharyngeal region, or odontogenic sources—a less commonly reported but clinically important etiology that is the focus of the present study. The predominant clinical features include persistent cephalalgia, temporomandibular joint discomfort, and impairment of cranial nerve function (3–5). The nonspecific clinical manifestations of atypical SBO frequently result in diagnostic challenges, including both misdiagnosis and failure to recognize the condition (6). This diagnostic delay can have severe consequences, as untreated infections may progress intracranially, potentially leading to life-threatening complications. These include meningitis, intracranial abscess formation (either subdural or parenchymal), cavernous sinus thrombosis, and other serious neurological sequelae that significantly increase morbidity and mortality risks (1, 6, 7).

This study presents a clinical case of odontogenic atypical SBO in a diabetic patient, providing a comprehensive analysis of diagnostic challenges and key differentiating characteristics. Through a systematic mini-review of relevant literature, we examine the pathophysiological mechanisms, current diagnostic approaches, therapeutic advancements, and strategies to prevent misdiagnosis of odontogenic atypical SBO. Our findings aim to enhance clinicians’ ability to recognize and appropriately manage this condition in its early stages, thereby minimizing diagnostic errors and improving patient outcomes.

## 2 Mini-review methods

A systematic narrative mini-review of the literature was conducted to synthesize the current evidence on atypical/odontogenic SBO, including its pathophysiology, clinical characteristics, diagnostic modalities, therapeutic strategies, and diagnostic pitfalls. The review methodology was designed to ensure comprehensiveness and scientific rigor, following the principles of narrative review for clinical case-associated literature synthesis (8).

### 2.1 Search databases and timeframe

Literature searches were performed in the following English and Chinese electronic databases (the primary databases for clinical and medical research) from January 2000 to December 2025:

- PubMed (https://pubmed.ncbi.nlm.nih.gov/)
- Embase (https://www.embase.com/)
- Web of Science (https://www.webofscience.com/)
- Cochrane Library (https://www.cochranelibrary.com/)
- China National Knowledge Infrastructure (CNKI, https://www.cnki.net/)
- Wanfang Data (https://www.wanfangdata.com.cn/)

The 25-year timeframe was selected to capture the most recent advancements in the diagnosis and management of SBO, while including foundational studies that established the current understanding of atypical/odontogenic SBO.

### 2.2 Search terms

Combined MeSH terms and free-text keywords were used for database searches, with no language restrictions (non-English studies were included if English abstracts were available). The core search terms, as listed below, were tailored to focus on odontogenic and atypical SBO and were combined using Boolean operators (*AND*/*OR*): *skull base osteomyelitis*, *atypical skull base osteomyelitis*, *central skull base osteomyelitis*, *non-otogenic skull base osteomyelitis*, *odontogenic infection*, *maxillofacial infection*, *skull base infection*, *diagnosis*, *misdiagnosis*, *treatment*, *diabetes mellitus.*

### 2.3 Study inclusion criteria

Studies meeting the following criteria were included in the mini-review: (1) Study type: Clinical case reports, case series, retrospective cohort studies, prospective studies, systematic reviews, meta-analyses, and narrative reviews focusing on SBO; (2) Study population: Human patients with atypical/central/non-otogenic SBO, with a specific focus on cases with odontogenic etiologies; (3) Content relevance: Studies reporting on the pathophysiology, clinical characteristics, diagnostic methods, therapeutic strategies, diagnostic pitfalls, or prognosis of atypical/odontogenic SBO; (4) Data availability: Studies with complete clinical, imaging, or therapeutic data (case reports/series with insufficient detail were excluded).

### 2.4 Study exclusion criteria

Studies were excluded if they met any of the following criteria: (1) Focus on otogenic SBO (the primary subtype of SBO, with distinct clinical features and etiology from atypical/odontogenic SBO); (2) Basic science studies (e.g., *in vitro* or animal experiments) without clinical relevance; (3) Studies on skull base infections of non-osteomyelitic origin (e.g., pure soft tissue abscess, meningitis without bony involvement); (4) Duplicate publications, conference abstracts, or unpublished data; (5) Studies with unextractable or incomplete key information (e.g., no clear diagnosis, missing therapeutic outcomes).

### 2.5 Study selection

The literature search and study selection process were independently performed by two authors (P.P. and C.-x.L.), with discrepancies resolved by a third senior author (Z.-c.G.) through consensus. The selection process followed a two-step approach: (1) Title and abstract screening: All retrieved citations were screened to exclude irrelevant studies based on the inclusion/exclusion criteria; (2) Full-text assessment: The full texts of potentially eligible studies were reviewed to confirm final eligibility.

## 3 Case presentation and investigations

**Figure 1:**
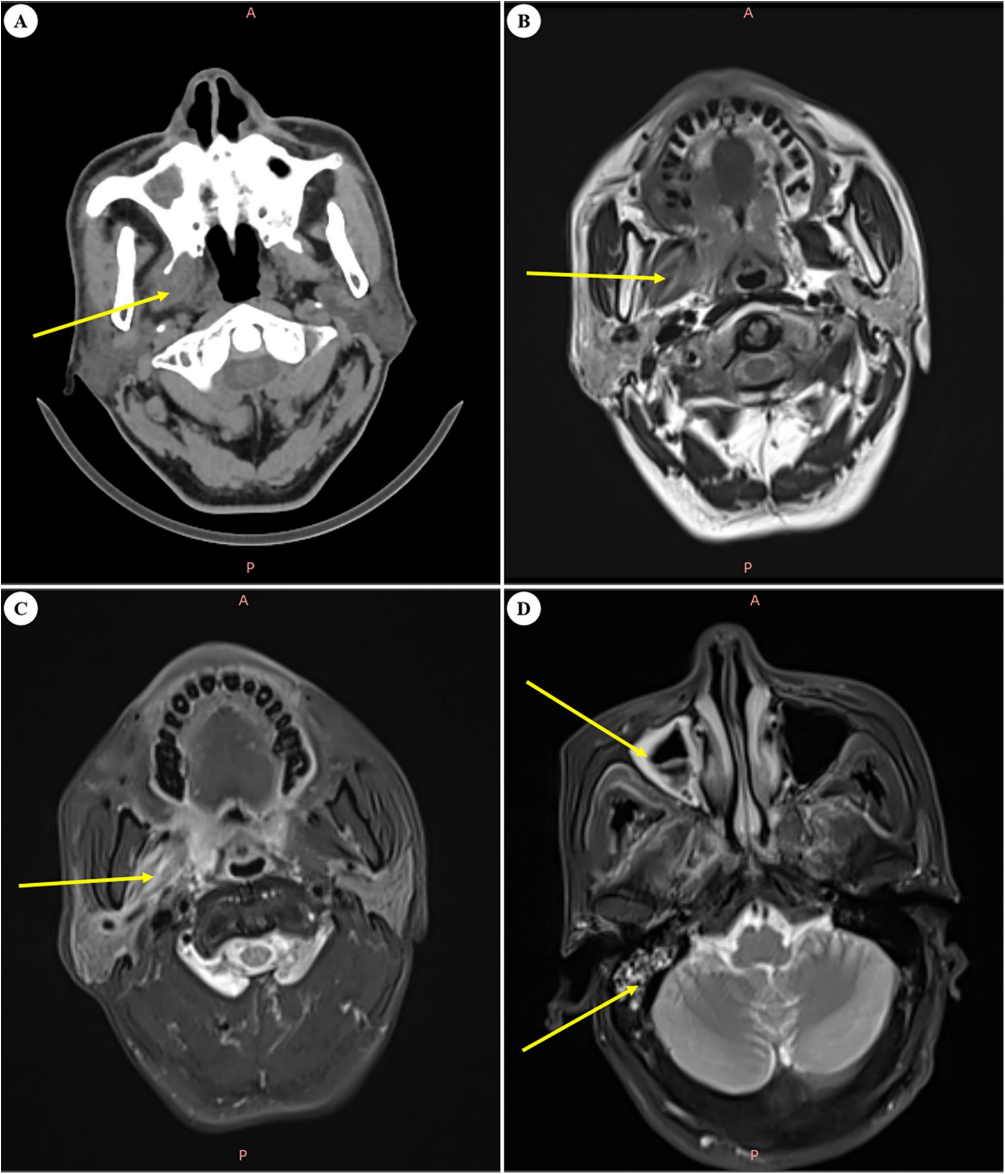
Imaging findings during the first hospitalization. **(A)** The right pterygoid muscle demonstrates increased volume with mild haziness in the adjacent adipose tissue (CT). **(B)** Edematous changes are observed in the right medial pterygoid muscle, exhibiting moderately elevated T2-weighted signal intensity in both the muscle and adjacent pharyngeal region (MRI). **(C)** Fluid-sensitive sequences reveal hyperintense signals localized to the right medial pterygoid muscle and pharyngeal space (MRI). **(D)** Concurrent inflammatory changes are noted, including right mastoiditis and right maxillary sinusitis (MRI).

A 57-year-old male with a two-year history of type 2 diabetes mellitus was admitted to a local hospital. The patient initially presented with a three-month history of idiopathic trismus, characterized by progressive restriction of mandibular mobility without identifiable precipitating factors. Based on the initial diagnosis of temporomandibular joint disorders, the patient underwent bilateral articular arthrocentesis using hyaluronic acid with supplemental physiotherapy. However, the therapeutic outcomes were unsatisfactory, with the patient's symptoms persisting and progressively deteriorating over time. Following clinical evaluation, this patient was transferred to our medical center for specialized craniofacial high-resolution spiral computed tomography (CT) scanning and magnetic resonance imaging (MRI). The CT and MRI findings demonstrated notable edema in the right medial pterygoid muscle along with opacification in the ipsilateral pharyngeal region, indicative of potential infectious pathology or abscess development (Figure 1). Subsequent cone beam CT (CBCT) imaging demonstrated irregular osseous sclerosis and elevated radiodensity in the region of the right maxillary tuberosity and posterior maxillary dentition (Supplementary Figure S1). These radiological findings prompted a revision of the initial diagnosis to “right maxillary sclerosing osteomyelitis complicated by right medial pterygoid myositis.” The therapeutic regimen consisted of intravenous administration of amoxicillin-clavulanate potassium (2.0 g every 12 h) for antimicrobial coverage, supplemented with adjunctive therapies including maxillofacial shortwave diathermy and photobiomodulation therapy (red light irradiation) over a 10-day treatment course. After the therapeutic intervention, the patient exhibited significant symptomatic relief and achieved full recovery of normal mandibular range of motion prior to being discharged from our medical facility.

However, approximately one month following hospital discharge, the patient experienced recurrence and progressive exacerbation of the aforementioned symptoms. These clinical manifestations primarily involved persistent pain localized to the right facial and auriculotemporal regions, concurrent with progressive trismus. The pain intensity escalated dramatically during the week preceding the subsequent medical consultation, reaching an intolerable level. Concurrently, the patient developed noticeable swelling in the preauricular region of the right ear, accompanied by marked worsening of mandibular movement restriction. Due to inadequate response to conservative management including local thermotherapy and oral medications (celecoxib and amoxicillin), the patient required hospital readmission for further evaluation and treatment.

At the second hospital admission, upon physical assessment, the patient presented with a body temperature of 36.4 °C, indicating no fever. Mild soft tissue swelling was observed in the pre-tragal region of the right ear, with normal cutaneous coloration and temperature, accompanied by localized tenderness. Both external auditory canals were clear without abnormal discharge. Evaluation of facial nerve function was within normal limits, corresponding to House-Brackmann grade I. Maximal mouth opening was restricted to less than the width of one finger, with midline alignment preserved. Intraoral examination revealed extensive supragingival calculus (+++) and subgingival deposits (++), generalized gingival erythema and edema, with bleeding on probing. The right maxillary second molar was absent, and the left mandibular second molar had been restored with a porcelain crown. The lower anterior teeth exhibited grade I to II mobility. These findings are consistent with a diagnosis of severe chronic periodontitis. Laboratory results included a white blood cell count of 9.63 × 10^9^/L, neutrophils accounting for 64.0% with an absolute count of 6.16 × 10^9^/L. Elevated inflammatory markers were noted: C-reactive protein at 30.4 mg/L, interleukin-6 at 20.4 pg/mL, and serum amyloid A at 89.59 mg/L. Procalcitonin levels were below 0.02 ng/mL. Glycated hemoglobin measured 7.34%, indicating moderately controlled glycemic status.

Follow-up CT scans obtained after readmission demonstrated notable radiographic alterations in the right maxillofacial region. The right maxillary molar area exhibited decreased alveolar bone density with irregular cortical margins and ill-defined adjacent tissue planes (Supplementary Figure S2A). Significant inflammatory changes were observed, including marked swelling of both medial and lateral pterygoid muscles on the right side, along with thickening of the nasopharyngeal wall soft tissues (Supplementary Figure S2B). The right parapharyngeal space and maxillofacial subcutaneous fat compartments displayed increased density (Supplementary Figure S2C). Bilateral maxillary sinusitis was present, with more pronounced involvement on the right side. Additionally, imaging findings consistent with right-sided mastoiditis were identified (Supplementary Figure S2D).

Comparative analysis with initial imaging studies revealed disease progression, characterized by worsening edema of the right medial pterygoid muscle and increased opacification of fat spaces. The right maxillary alveolar bone showed further demineralization, while the right parapharyngeal and maxillofacial subcutaneous fat spaces demonstrated greater density. Subsequent CT evaluation during disease advancement revealed heterogeneous bone density reduction affecting the right mandibular condyle and zygomatic arch (Supplementary Figure S3A). Confirmatory MRI demonstrated newly developed bone marrow edema in these same anatomical structures (Supplementary Figure S3B).

## 4 Diagnosis, treatment, outcome and follow-up

The final diagnoses, incorporating comprehensive systemic evaluation and clinical findings, revealed two primary conditions: (1) odontogenic atypical osteomyelitis of the skull base (originating from chronic periodontitis and maxillary alveolar bone infection) and (2) severe chronic periodontitis. Upon hospital admission, immediate intravenous administration of cefotaxime (2.0 g, bid) was initiated alongside adjunctive local physical therapy. The patient's concurrent chronic pulmonary disease precluded the utilization of hyperbaric oxygen therapy (HBOT).

Clinical progression during the first week of hospitalization demonstrated exacerbation of right temporal swelling and the emergence of right ocular abduction dysfunction, suggesting potential cavernous sinus involvement. Surgical intervention revealed substantial purulent accumulation within the right deep temporal space, which was thoroughly evacuated and subsequently subjected to microbiological culture and antimicrobial susceptibility testing. The specific surgical steps were conducted as follows: Following the induction of general anesthesia, a conventional preauricular incision was made on the right side. The dissection proceeded layer by layer through the skin, subcutaneous fat, and muscle planes to reach the deep temporal and infratemporal regions. Using blunt dissection techniques, the abscess cavity was fully exposed, and approximately 15 mL of malodorous grayish-white purulent material was evacuated. The operative site was thoroughly irrigated with normal saline solution until the returning fluid appeared clear. For histopathological analysis, a sample of temporal periosteum and adjoining tissue was resected from the wall of the cavity. After achieving careful hemostasis, a vacuum sealing drainage (VSD) system was positioned within the cavity to ensure ongoing drainage, and the wound was subsequently closed. Microbiological analysis identified *Streptococcus pneumoniae* as the causative pathogen, demonstrating susceptibility to multiple agents including amoxicillin-clavulanate, cefuroxime, cefotaxime, clindamycin, and linezolid, while exhibiting resistance to erythromycin. The treatment protocol was consequently modified to incorporate targeted, culture-directed antibiotic therapy at appropriate therapeutic doses.

**Figure 2:**
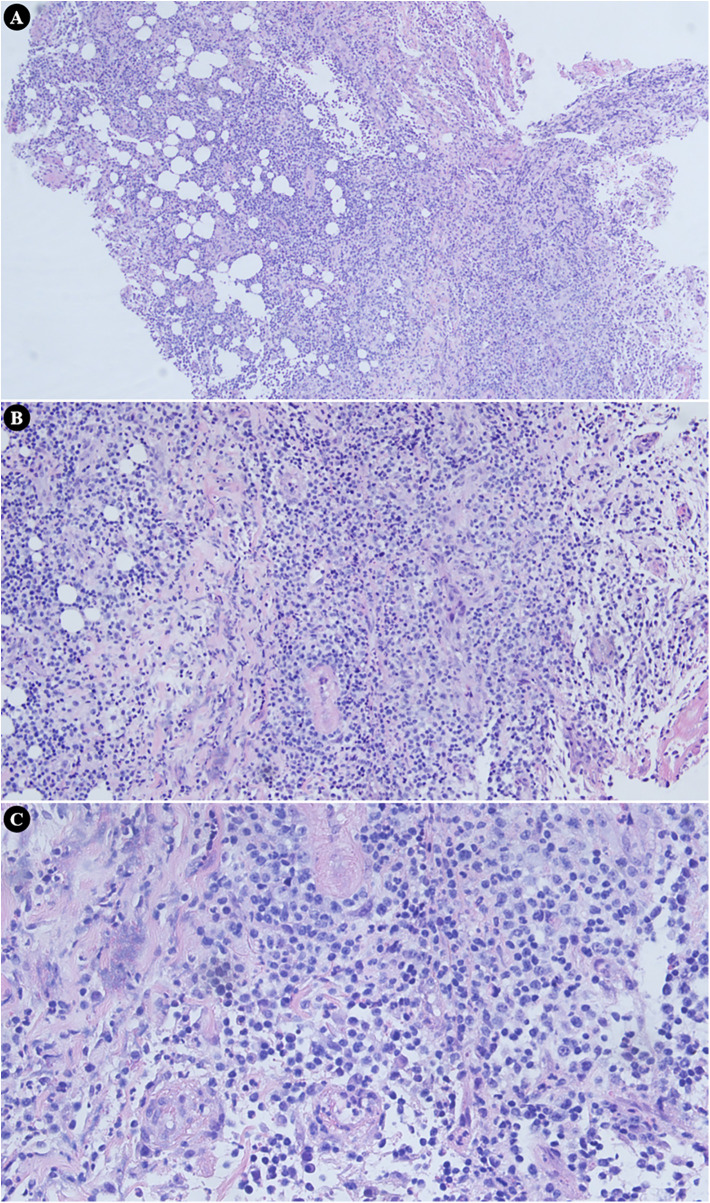
Microscopic analysis of the surgically resected deep temporal tissue from the right side revealed widespread infiltration by neutrophils along with necrotic cellular debris, establishing a diagnosis of acute suppurative inflammation with no indications of malignant change. **(A)** Original magnification × 100. **(B)** Original magnification × 200. **(C)** Original magnification × 400.

Histopathological examination of the excised right deep temporal tissue specimen demonstrated extensive neutrophilic infiltration and necrotic debris, confirming acute suppurative inflammation without evidence of malignant transformation (Figure 2). Following surgical drainage and tailored antimicrobial therapy, the patient exhibited marked improvement in right maxillofacial swelling, pain symptoms, and trismus. Partial restoration of right eye abduction function was achieved. Serial measurements of inflammatory markers exhibited a consistent downward trend following the surgical procedure, as outlined in Supplementary Table S1. At the 12-month follow-up assessment, the patient maintained satisfactory clinical status, with radiographic imaging demonstrating complete resolution of the skull base and deep temporal infectious foci, and no signs of disease recurrence were observed.

## 5 Discussion

### 5.1 Overview of SBO etiopathogenesis

SBO represents a severe infectious condition affecting the osseous structures of the cranial base, including the temporal, sphenoid, and occipital bones, with bacterial or fungal pathogens being the primary etiological agents. The otogenic variant typically disseminates through anatomical pathways such as fissures of Santorini, tympanic cavity, mastoid processes, and petrous apex, whereas nasopharyngeal or odontogenic-originating infections may involve the skull base and dural sinuses via the parapharyngeal space, pterygopalatine fossa, and masticator space (9)—the key dissemination pathway in the present odontogenic SBO case. Among bacterial pathogens, *Pseudomonas aeruginosa* predominates in otogenic SBO, though other organisms including *Staphylococcus aureus*, coagulase-negative staphylococci, *Streptococcus pneumoniae* (the pathogen in our case), and anaerobic bacteria have been implicated in odontogenic atypical SBO. Immunocompromised individuals and diabetic patients demonstrate increased susceptibility to fungal pathogens such as Aspergillus, Mucor, and Candida species (10).

The pathophysiological mechanisms underlying disease progression in high-risk populations (diabetics, elderly individuals, immunocompromised patients, and those with chronic sinusitis or uncontrolled oromaxillofacial infections) involve neutrophil dysfunction, compromised osseous vascular supply, and impaired tissue repair capacity (11). The clinical course is characteristically indolent yet progressive, with potential for dural invasion and intracranial extension if untreated, leading to life-threatening complications including meningitis, cerebral abscess formation, and venous sinus thrombosis, associated with mortality rates approaching 10%–20% (5). Notably, approximately 30% of treated patients experience permanent cranial neuropathies (12).

A distinct clinical entity, termed atypical SBO, does not originate from external auditory canal infections. These atypical presentations, predominantly affecting central skull base regions including the clivus and sphenoid body, are categorized as “central” or “non-otogenic” SBO. Potential infectious sources may include nasopharyngeal foci, sinus infections, dental pathologies (the primary etiology in this study), or postoperative complications following head and neck procedures (1, 2, 13). In contrast to conventional presentations, atypical cases frequently present without characteristic otologic symptoms such as otalgia or otorrhea. Instead, the clinical picture is typically dominated by persistent headaches or intractable orofacial pain as primary manifestations. The nonspecific nature of these symptoms frequently necessitates differential diagnosis from intracranial neoplastic lesions during initial evaluation, significantly complicating the diagnostic process.

### 5.2 Clinical characteristics and diagnostic approach

The clinical presentation of atypical SBO typically follows an indolent course with nonspecific initial manifestations. In the present odontogenic SBO case, the patient exhibited neither significant external auditory canal discharge nor overt otologic findings, instead presenting primarily with temporomandibular joint pain and trismus—symptoms closely mimicking temporomandibular joint disorders, the primary reason for the initial misdiagnosis. These atypical features frequently lead to misdiagnosis as more common conditions including otitis media, facial neuritis, or temporomandibular joint disorders, particularly when the infection originates from odontogenic sources with no overt otologic involvement.

Diagnostic imaging plays a pivotal role in SBO evaluation, though interpretation challenges exist. Conventional skull CT in early stages may yield negative results or demonstrate only nonspecific alterations. MRI demonstrates superior sensitivity for detecting early bone marrow edema and soft tissue involvement, typically revealing T1-hypointensity, T2-hyperintensity, and contrast enhancement in affected osseous structures, while also delineating soft tissue abscess extent and fascial space spread patterns—critical for identifying the spread of odontogenic infections to the skull base. CT proves valuable for assessing progressive bone destruction and sequestrum formation (11, 14, 15). Radionuclide imaging using technetium-99 m three-phase bone scanning exhibits exceptional sensitivity (approaching 100%) for early SBO detection (15, 16). However, its limited specificity and persistent positivity post-treatment render it unsuitable for therapeutic monitoring. Emerging evidence suggests potential utility of 2-deoxy-2-fluorodeoxyglucose positron emission tomography/CT (18F-FDG PET/CT) for both diagnosis and treatment response assessment in SBO, though widespread adoption remains constrained by equipment availability and operator expertise (3, 17).

Notably, histopathological examination and microbiological confirmation of biopsied lesional tissue constitute the diagnostic gold standard. Given the considerable imaging overlap between SBO and various conditions—including skull base masses (such as nasopharyngeal malignancies or metastatic tumorss), idiopathic skull base fibrosis, and granulomatous diseases (e.g., granulomatosis with polyangiitis)—clinicians must maintain heightened suspicion. When imaging cannot exclude malignancy or empirical therapy proves ineffective, prompt biopsy becomes imperative for definitive diagnosis. In our reported odontogenic SBO case, the initial diagnosis of “sclerosing osteomyelitis” relied solely on maxillary imaging findings. However, subsequent disease recurrence necessitated surgical exploration and biopsy, ultimately confirming skull base infection secondary to odontogenic spread. This underscores the diagnostic value of early tissue sampling and microbiological analysis in equivocal cases, facilitating accurate differentiation and guiding appropriate therapeutic interventions.

### 5.3 Treatment strategy and prognosis

The core treatment for SBO is a long-term and adequate antimicrobial therapy, usually lasting 6–20 weeks (18). For odontogenic atypical SBO, empirical antibiotic regimens should target oral flora pathogens (e.g., *Streptococcus* species, *Staphylococcus aureus*, anaerobic bacteria)—in contrast to otogenic SBO, where *Pseudomonas aeruginosa* is the primary target (11, 19). Fluoroquinolones, such as ciprofloxacin, may be used as adjunctive therapy for bone penetration, while beta-lactam/beta-lactamase inhibitor combinations (e.g., amoxicillin-clavulanate) or cephalosporins (e.g., cefotaxime) are preferred for primary coverage of odontogenic bacterial pathogens. Immunocompromised patients often require adjunctive antifungal agents—such as fluconazole or amphotericin B and itraconazole for Aspergillus/Mucor infections—guided by microbiological confirmation. In the initial stage, intravenous administration is commonly used. A retrospective study conducted by Johnson and colleague involving 42 cases of central SBO demonstrated that approximately 55% of patients required intravenous medication for 6–9 weeks (2). Following clinical and radiological improvement, these patients could transition to oral maintenance therapy, with the total treatment duration typically extending beyond 3 months (2). Concurrently, glycemic control in diabetic patients and immune support in immunodeficient individuals are essential to enhance therapeutic outcomes (20)—a key management step in our diabetic patient with odontogenic SBO.

Surgical intervention in SBO is primarily reserved for debridement of necrotic bone, drainage of abscesses, decompression of intracranial structures, and procurement of biopsy specimens (20). Approximately 43% of patients undergo surgery alongside antibiotics, with procedures including mastoidectomy, drainage, excision of nasopharyngeal granulation tissue, and skull base decompression (21). For odontogenic atypical SBO, surgical intervention also includes drainage of deep facial space abscesses (e.g., deep temporal space, masticator space) and debridement of infected odontogenic bone (e.g., maxillary alveolar bone), as performed in our case. The objective of surgery is to reduce infectious burden and improve regional perfusion rather than to eradicate infection outright. Indications for operative management should be determined through multidisciplinary evaluation (22).

In recent years, HBOT has gained recognition as an adjuvant treatment. HBO elevates tissue oxygen levels, enhances macrophage-mediated bacterial clearance, and supports osteoneogenesis (23). A retrospective analysis spanning 2006–2023 demonstrated symptomatic and imaging improvement in 87% of patients receiving adjunctive HBOT (24). Thus, HBOT is recommended early in the management of refractory cases, extensive disease, or diabetic patients—though it was not feasible in our case due to the patient's chronic pulmonary comorbidity. Prognosis is strongly influenced by timely diagnosis, appropriate intervention, and host factors. Studies report an 18-month survival rate of 90.5% with aggressive treatment, although mortality remains elevated in diabetics (20%–30%) (25), and roughly 30% of survivors experience persistent cranial neuropathies (26). Standardized early treatment is therefore critical for reducing both mortality and long-term morbidity, particularly in odontogenic SBO cases where early diagnosis is often delayed due to nonspecific symptoms.

### 5.4 Analysis of the reasons for misdiagnosis

SBO, especially its atypical form, is frequently misdiagnosed due to its rarity and diverse clinical presentations. A notable feature is the frequent absence of classic otogenic symptoms—the primary distinguishing feature from otogenic SBO. In this odontogenic SBO case report, the primary manifestations included temporomandibular joint discomfort without systemic signs of infection such as fever, which closely mimicked temporomandibular joint disorders and led to the initial misdiagnosis. The initial assessment failed to adequately consider the patient's history of diabetes, a key risk factor for SBO, contributing to a delayed diagnosis. Furthermore, imaging modalities exhibit limited sensitivity. Early-stage SBO may present without distinctive features on conventional CT scans (13), leading to oversight or misinterpretation. Although imaging in this case indicated sclerotic changes in the maxillary bone (the odontogenic source), the involvement of the skull base was not clearly delineated in the early stage, resulting in a diagnosis confined to localized osteomyelitis rather than the more severe skull base involvement. Additionally, radiographic findings can mimic other pathologies. The midline crossover of lesions affecting the clivus or occipital bone may be mistaken for nasopharyngeal carcinoma or bony neoplasms. Chronic sclerosing osteomyelitis (the initial diagnosis in our case) is also susceptible to being misidentified as osteophyte formation. Finally, the absence of definitive microbiological evidence poses a significant challenge. Biopsy of the skull base is technically demanding, and clinicians often resort to empirical antibiotic therapy. Early pathogen identification through biopsy and subsequent targeted antimicrobial treatment could help reduce diagnostic errors, particularly in odontogenic SBO cases with non-typical pathogens.

### 5.5 Prevention and countermeasures

To address the diagnostic challenges associated with atypical SBO, a comprehensive clinical approach should be implemented focusing on heightened clinical suspicion and standardized diagnostic protocols. The following strategies are recommended (Supplementary Table S2).

The standardized protocols for long-term monitoring and therapeutic management must be implemented. Given the protracted clinical course and high recurrence propensity of SBO, rigorous adherence monitoring should be complemented by periodic imaging surveillance and inflammatory marker assessment. For diabetic patients, collaborative glycemic optimization with endocrinology specialists is essential to enhance host defense mechanisms—a critical step for odontogenic SBO patients with diabetes, as seen in our case. High-risk individuals (e.g., diabetics with chronic periodontitis or odontogenic infections) warrant extended surveillance for a minimum of 12 months post-treatment to detect early recurrence indicators.

Furthermore, medical education programs should emphasize SBO pathophysiology through case-based learning, particularly highlighting diagnostic pitfalls in odontogenic atypical presentations—a less commonly reported subtype that is often overlooked by clinicians. Primary care providers and oral and maxillofacial specialists require enhanced training in recognizing rare, severe infections originating from odontogenic sources and spreading to the skull base. Multidisciplinary consultation pathways (oral and maxillofacial surgery, otorhinolaryngology, neurology, radiology) should be established to ensure prompt specialist referral for complex cases, thereby minimizing diagnostic delays and inappropriate management.

## 6 Limitations

This study is constrained by its single-case design, necessitating cautious interpretation of the findings and conclusions due to limited generalizability to broader populations of odontogenic atypical SBO patients. Diagnostic and therapeutic decisions were guided by the protocols and clinical experience of a single institution. Furthermore, fungal culture was not conducted on the collected specimen, potentially missing concurrent fungal or mixed fungal-bacterial infections—a limitation that should be addressed in future clinical practice for SBO patients. Additionally, the mini-review is a narrative synthesis (not a systematic review/meta-analysis) due to the limited number of studies focusing specifically on odontogenic atypical SBO (most studies focus on otogenic SBO), which limits the ability to perform a quantitative meta-analysis. Future research should involve multicenter, large-scale prospective studies to validate and refine standardized diagnostic and therapeutic strategies for odontogenic atypical SBO, and a systematic review with meta-analysis as more clinical data on this rare subtype becomes available.

## 7 Concluding remarks

Although atypical SBO is rare, odontogenic atypical SBO is an even less commonly reported subtype that poses unique diagnostic challenges due to its nonspecific clinical manifestations and lack of classic otogenic features. Delayed diagnosis and treatment can lead to serious, life-threatening intracranial complications. This report describes a case of odontogenic atypical SBO initially misdiagnosed as temporomandibular joint disorders due to non-specific clinical presentations, where definitive diagnosis and successful management were ultimately achieved through advanced imaging and multidisciplinary team (MDT) approaches. Clinical observations suggest that persistent craniofacial pain, temporomandibular joint dysfunction, or cranial nerve impairment in middle-aged to elderly individuals or immunocompromised hosts (particularly diabetics), with a history of chronic odontogenic or maxillofacial infections, should raise high suspicion for odontogenic atypical SBO. Furthermore, patients exhibiting refractory headaches, facial pain, or temporomandibular symptoms unresponsive to standard therapies warrant prompt imaging evaluation (MRI + CT) to prevent diagnostic oversight of this potentially devastating condition.

Accurate diagnosis of odontogenic atypical SBO necessitates both clinical suspicion and systematic exclusion of alternative diagnoses. Confirmation requires integration of characteristic radiological findings (bone marrow edema on MRI, bony destruction on CT) with microbiological and histopathological evidence. Therapeutic strategies should incorporate tailored antimicrobial regimens (targeting oral flora pathogens) with adequate duration, potentially augmented by surgical debridement/drainage (of both odontogenic sources and skull base/ deep facial space abscesses) and adjunctive HBOT when indicated. Concurrent management of predisposing conditions (e.g., glycemic control in diabetes, treatment of chronic periodontitis) is essential, as these comorbidities significantly influence treatment outcomes.

In conclusion, odontogenic atypical SBO presents unique diagnostic challenges due to its subtle onset, odontogenic etiology, and frequent mimicry of more common disorders. Implementation of three key strategies—heightened clinical awareness in at-risk populations with odontogenic infections; establishment of a comprehensive diagnostic pathway combining advanced imaging, microbiological/histopathological analysis, and MDT input; and adherence to personalized antimicrobial and surgical protocols with rigorous long-term follow-up—can substantially decrease rates of diagnostic errors, inappropriate treatment, and permanent neurological sequelae for this rare and severe condition.

## Supplementary material

Supplementary Table S1 Postoperative dynamic changes in inflammatory markers.

Supplementary Table S2 Prevention and countermeasures against osteomyelitis of skull base.

Supplementary Figure S1 CBCT reveals abnormal bone sclerosis with increased radiographic density localized to the right maxillary tuberosity and adjacent posterior maxillary dentition. The imaging findings showed irregular bony changes characterized by elevated mineralization in this anatomical region. **A.** Axial plane. **B.** Coronal plane. **C.** Sagittal plane. **D.** Three-dimensional reconstructive view.

Supplementary Figure S2 CT re-evaluation findings from the second admission. **A.** The assessment shows reduced alveolar bone density with irregular borders and poorly demarcated adjacent spaces in the right maxillary molar area. **B.** Significant swelling is noted in the medial and lateral pterygoid muscles on the right, along with thickening of the peri-nasopharyngeal soft tissues. **C.** Increased radiodensity is observed in the right parapharyngeal and maxillofacial subcutaneous fat layers. **D.** Bilateral maxillary sinus inflammation is present, with more severe involvement on the right. These imaging features align with right mastoiditis. Comparative review indicates disease advancement, including more pronounced medial pterygoid muscle enlargement and broader opacification of adipose compartments compared to earlier imaging. Additional alveolar demineralization is noted. New observations include intensified soft tissue infiltration and elevated density within the involved fat spaces.

Supplementary Figure S3 Disease recurrence and progression. **A.** Imaging reveals newly apparent bony alterations in the right mandibular condyle and zygomatic arch, exhibiting uneven bone density with slight reduction. **B.** Marrow edema is visible within these regions, indicating ongoing inflammatory activity.
