# Supplementary material for: Odontogenic, atypical skull-base osteomyelitis: diagnostic pitfalls and therapeutic insights—a case report and mini-review
Source: Front Oral Health. 2026 Apr 22;7:1789196. doi: 10.3389/froh.2026.1789196 (PMC13145246; doi:10.3389/froh.2026.1789196)
Supplement: Supplementary Table S1 — Postoperative dynamic changes in inflammatory markers. [file table1.doc]

*****Supplementary Table 1. Postoperative dynamic changes in inflammatory markers.*****

| Time point | C-reactive protein (mg/L) | Interleukin-6 (pg/mL) | Procalcitonin (ng/mL) | White blood cell count (×10⁹/L) |
| --- | --- | --- | --- | --- |
| 1 day before surgery | 38.6 | 12.10 | <0.02 | 11.42 |
| Postoperative Day 1 | 21.7 | 9.81 | 0.03 | 8.05 |
| Postoperative Day 4 | 31.3 | 15.60 | 0.05 | 9.26 |
| Postoperative Day 5 | 27.0 | 8.87 | 0.08 | 11.59 |
| Postoperative Day 6 | 23.0 | 4.52 | 0.06 | 10.75 |
| Postoperative Day 7 | 19.9 | 8.73 | 0.06 | 11.28 |
| Postoperative Day 11 | ****<5.0**** | ****5.69**** | ****<0.02**** | ****9.56**** |

*Note: Normal reference ranges are as follows: C-reactive protein < 10 mg/L; Interleukin-6 < 7 pg/mL; Procalcitonin < 0.05 ng/mL; White blood cell count 3.5–9.5 ×10⁹/L. Bold values indicate a return to or near-normal levels.
