# Supplementary material for: Odontogenic, atypical skull-base osteomyelitis: diagnostic pitfalls and therapeutic insights—a case report and mini-review
Source: Front Oral Health. 2026 Apr 22;7:1789196. doi: 10.3389/froh.2026.1789196 (PMC13145246; doi:10.3389/froh.2026.1789196)
Supplement: Supplementary Table S2 — Prevention and countermeasures against osteomyelitis of skull base. [file table2.doc]

| Supplementary Table 2. Prevention and countermeasures against osteomyelitis of skull base | |
| --- | --- |
| Subheadings | Description |
| Enhanced Clinical Vigilance | Particular attention should be paid to high-risk populations and atypical presentations. Diabetic elderly patients presenting with persistent craniofacial pain, cranial neuropathies, or trismus – even in the absence of overt otologic symptoms – warrant consideration of SBO in the differential diagnosis. Conversely, younger patients without conventional risk factors demonstrating refractory symptoms of unclear etiology should not be dismissed based solely on their age or metabolic status. |
| Advanced Imaging Protocol | For cases with prolonged or recurrent localized infections, prompt radiological assessment of the skull base is imperative. A multimodal imaging approach combining high-resolution temporal bone CT with contrast-enhanced MRI is recommended for suspected cases to optimize diagnostic yield. When indicated, supplementary nuclear medicine studies (bone scintigraphy or PET-CT) may be employed to detect occult osseous involvement. |
| Microbiological Confirmation | In cases where malignancy cannot be excluded or the infectious source remains indeterminate, early tissue sampling is crucial. This may involve targeted biopsy of suspicious lesions using either conventional surgical or minimally invasive techniques to obtain adequate specimens for histopathological and microbiological analysis. Biopsy serves dual diagnostic purposes: it enables definitive tumor differentiation while facilitating pathogen identification through microbial culture and histopathological analysis, thereby informing targeted therapeutic strategies. Particularly in cases refractory to empirical therapy, indiscriminate antibiotic rotation should be avoided; rather, treatment modification should be based on etiological confirmation through proper specimen collection. |
| Multidisciplinary Management | Given the diagnostic complexity and heterogeneous presentation of SBO, a collaborative approach involving otolaryngology, maxillofacial surgery, neurosurgery, and infectious disease specialists is essential. As evidenced in current literature, optimal management requires coordinated multidisciplinary evaluation to formulate individualized treatment strategies. |
