# Supplementary figures and images for: Odontogenic, atypical skull-base osteomyelitis: diagnostic pitfalls and therapeutic insights—a case report and mini-review
Source: Front Oral Health. 2026 Apr 22;7:1789196. doi: 10.3389/froh.2026.1789196 (PMC13145246; doi:10.3389/froh.2026.1789196)

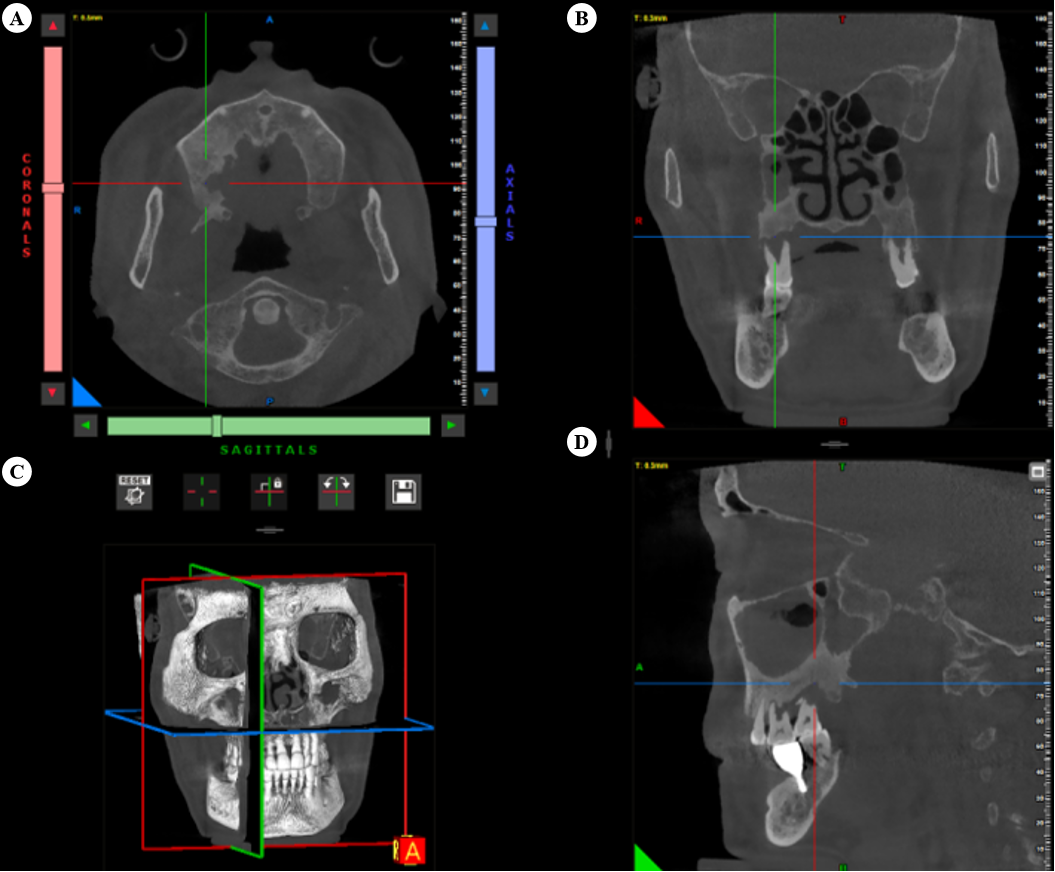

Supplement: Supplementary Figure S1 — CBCT reveals abnormal bone sclerosis with increased radiographic density localized to the right maxillary tuberosity and adjacent posterior maxillary dentition. The imaging findings showed irregular bony changes characterized by elevated mineralization in this anatomical region. A. Axial plane. B. Coronal plane. C. Sagittal plane. D. Three-dimensional reconstructive view. [file image1.tif]

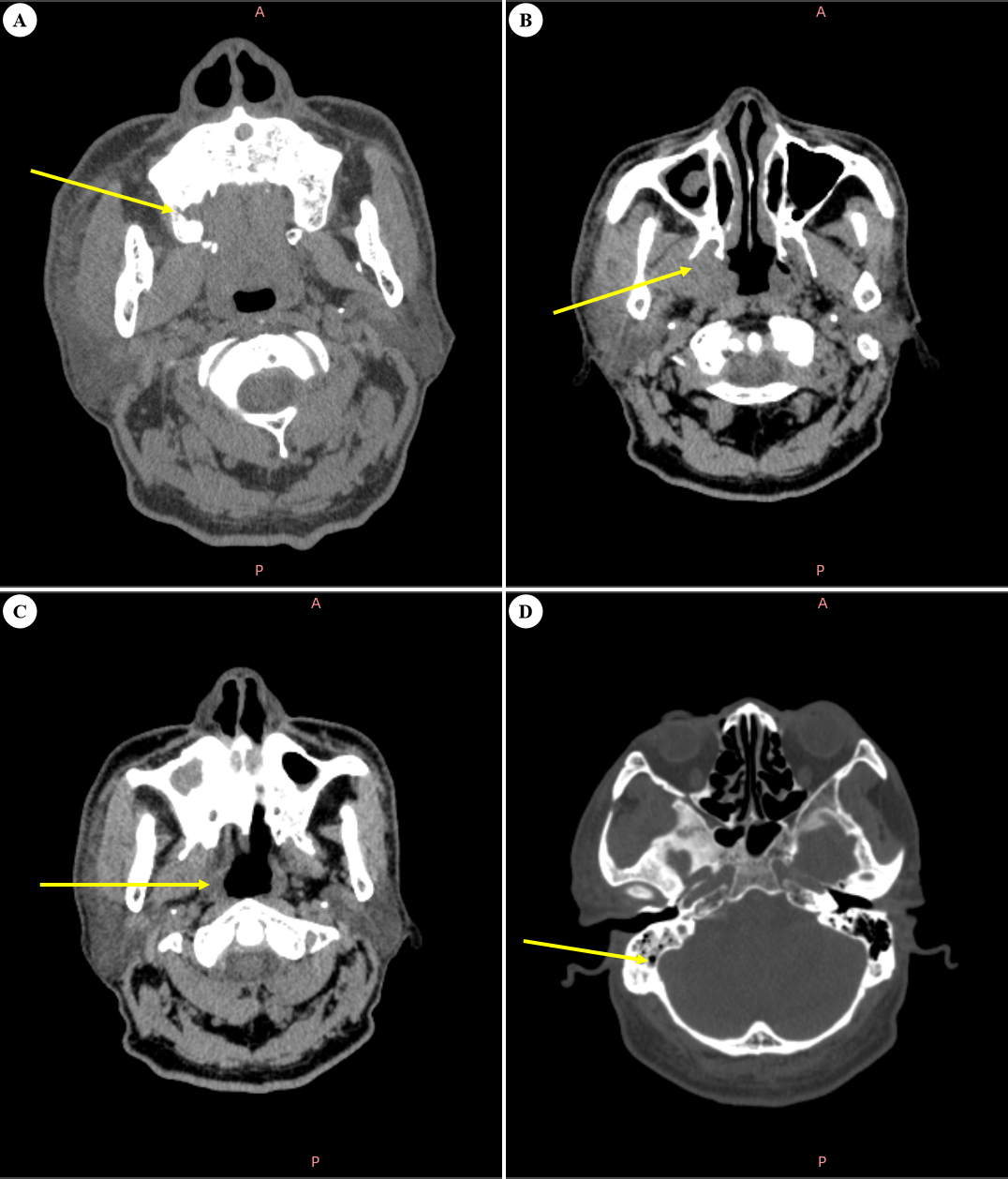

Supplement: Supplementary Figure S2 — CT re-evaluation findings from the second admission. A. The assessment shows reduced alveolar bone density with irregular borders and poorly demarcated adjacent spaces in the right maxillary molar area. B. Significant swelling is noted in the medial and lateral pterygoid muscles on the right, along with thickening of the peri-nasopharyngeal soft tissues. C. Increased radiodensity is observed in the right parapharyngeal and maxillofacial subcutaneous fat layers. D. Bilateral maxillary sinus inflammation is present, with more severe involvement on the right. These imaging features align with right mastoiditis. Comparative review indicates disease advancement, including more pronounced medial pterygoid muscle enlargement and broader opacification of adipose compartments compared to earlier imaging. Additional alveolar demineralization is noted. New observations include intensified soft tissue infiltration and elevated density within the involved fat spaces. [file image2.tif]

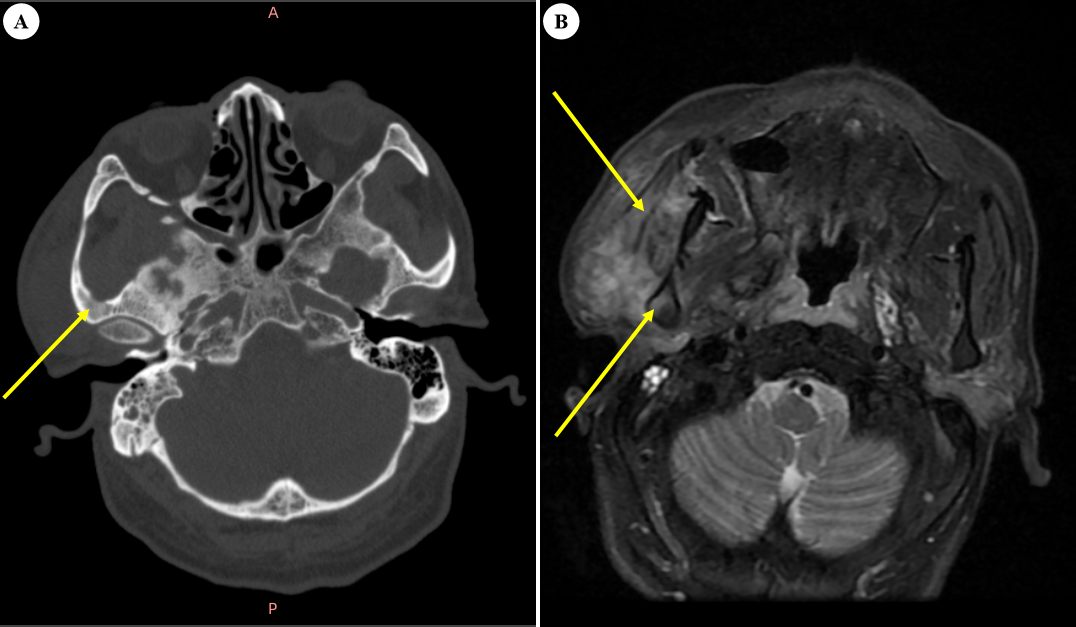

Supplement: Supplementary Figure S3 — Disease recurrence and progression. A. Imaging reveals newly apparent bony alterations in the right mandibular condyle and zygomatic arch, exhibiting uneven bone density with slight reduction. B. Marrow edema is visible within these regions, indicating ongoing inflammatory activity. [file image3.tif]
